# Personal

**Published:** 1906-04

**Authors:** 


the present legislature may deem wise to enact, or no matter what it does enact, future legislatures will be obliged to meet the propositions that confront them and to deal with them as seems best at the time, irrespective of what their predecessors may have done under similar conditions.

The osteopaths are likely to be just as clamorous in the future as in the past, until they are given to understand in plain terms that theirs is not a scientific body, and no legal enactment can make it so; that if they wish to be called doctors they must take the prescribed course of study and obtain the degree in the same manner as other American citizens are obliged to do; and, finally, no matter what other commonwealths have done or may do, the Empire State will not lower its educational standards, nor grant special privileges, to any group of its citizens no matter how respectable, nor how honest their intentions.

As far as the new medical bill is concerned we can trust the legislature to gain all the light possible on the educational question in this state, and then to do the right thing—to act with equity toward the ten thousand physicians who have been commissioned by law to protect seven millions of people against the ravages of preventable disease.

---

It has recently been our privilege to sample the oil and olives produced by Mr. T. W. Chapman formerly of San Fernando, Los Angeles County, California, now residing in this city and demonstrating the good qualities of both at his business office, 46 West Huron Street. Physicians throughout the world recogonize the high medicinal and food values of strictly pure olive oil. It is worth while to know where to get the genuine at all times, and we take pleasure in calling attention to Mr. Chapman's advertisement elsewhere in this issue.

---

### PERSONAL.

Dr. Lee Masten Francis, of Buffalo,, announces the removal, on the first of May, of his office and residence to 482 Delaware Avenue. Hours 9 to 1. Afternoons by appointment.

---

Dr. William Lee Howard, of Baltimore, will remove May 1, 1906, to "Mossfell", Westboro, Mass., where he will devote his time principally to literature. We hope to be able to present our readers with occasional contributions from Dr. Howard's facile pen.

---

Dr. Eli H. Long, professor of Materia Medica and Therapeutics in the University of Buffalo, was elected a member of the judicial council of the Association of American Medical Colleges at its sixteenth annual meeting held at Pittsburg, March 19, 1906.

---

DR. JOHN H. PRYOR, formerly of this city and late of Sarnac Lake, has returned to Buffalo and resumed the practice of his profession. His office and residence is at 26 Linwood Avenue. Dr. Pryor will be welcomed back to his old home by a very large clientele, and by numerous professional friends. Hours, 9 to 10, and 2 to 4.

---

DR. GEORGE BEN JOHNSTON, of Richmond, was the guest of Dr. Roswell Park, 510 Delaware Avenue, Buffalo, Tuesday, April 3, 1906. Dr. Park invited a company of men to meet Dr. Johnston at dinner, and afterward Dr. Johnston read a paper before the Buffalo Academy of Medicine, entitled "Fibroid Tumors of the Uterus Complicated by Pregnancy, with a report of Case."

---

DR. MARTHA F. CAUL, of Buffalo, who has been in Porto Rico during the past winter, has returned to her home, 87 West Genesee Street and resumed her professional practice.

---

DR. L. S. MCMURTRY, of Louisville, president of the American Medical Association, was the guest of honor at the annual meeting of the Louisville Academy of Medicine, March 7, 1906.

---

DR. CHARLES A. L. REED, of Cincinnati, was the guest of honor at the meeting of the Saint Louis Obstetrical and Gynecological Society, March 15, 1906, at which time he read a paper before the society on "The Surgical Treatment of So-called Idiopathic Dilatation of the Stomach."

Dr. Reed was accompanied to Saint Louis by Dr. L. S. McMurtry, Dr. H. H. Grant, and Dr. Arch. Dixon, of Kentucky. The visitors inspected the new City Hospital and were the guests of the Surgeon in charge, Dr. John Young Brown, during their stay in Saint Louis.

---

DR. ROBERT T. MORRIS, of New York, delivered a lecture March 14, 1906, before the Linnean Society of New York City at the American Museum of Natural History, the subject being, "A Naturalist's Camping Trip to Hudson's Bay." Dr. Morris spent the season in the Hudson's Bay region last year where he had an interesting and novel experience which he relates in a fascinating manner.

